# A clinical algorithm for same-day HIV treatment initiation in settings with high TB symptom prevalence in South Africa: The SLATE II individually randomized clinical trial

**DOI:** 10.1371/journal.pmed.1003226

**Published:** 2020-08-27

**Authors:** Mhairi Maskew, Alana T. Brennan, Matthew P. Fox, Lungisile Vezi, Willem D. F. Venter, Peter Ehrenkranz, Sydney Rosen

**Affiliations:** 1 Health Economics and Epidemiology Research Office, Department of Internal Medicine, School of Clinical Medicine, Faculty of Health Sciences, University of the Witwatersrand, Johannesburg, South Africa; 2 Department of Global Health, Boston University School of Public Health, Boston, Massachusetts, United States of America; 3 Department of Epidemiology, Boston University School of Public Health, Boston, Massachusetts, United States of America; 4 Ezintsha, Wits Reproductive Health and HIV Institute, Department of Internal Medicine, School of Clinical Medicine, Faculty of Health Sciences, University of the Witwatersrand, Johannesburg, South Africa; 5 Bill & Melinda Gates Foundation, Seattle, Washington, United States of America; University of Southampton, UNITED KINGDOM

## Abstract

**Background:**

Many countries encourage same-day initiation of antiretroviral therapy (ART), but evidence on eligibility for same-day initiation, how best to implement it, and its impact on outcomes remains scarce. Building on the Simplified Algorithm for Treatment Eligibility (SLATE) I trial, in which nearly half of participants were ineligible for same-day initiation mainly because of TB symptoms, the study evaluated the revised SLATE II algorithm, which allowed same-day initiation for patients with mild TB symptoms and other less serious reasons for delay.

**Methods and findings:**

SLATE II was a nonblinded, 1:1 individually randomized pragmatic trial at three primary healthcare clinics in Johannesburg, South Africa. It randomized adult patients presenting for an HIV test or any HIV care but not yet on ART. Intervention arm patients were assessed with a symptom screen, medical history, brief physical examination, and readiness questionnaire to distinguish between patients eligible for immediate ART dispensing and those requiring further care before initiation. Standard arm patients received usual care. Follow-up was by review of routine clinic records. Primary outcomes were (1) ART initiation in ≤7 days and (2) ART initiation in ≤28 days and retention in care at 8 months (composite outcome). From 14 March to 18 September 2018, 593 adult HIV+, nonpregnant patients were enrolled (median interquartile range [IQR] age 35 [29–43]; 63% (*n* = 373) female; median CD4 count 293 [133–487]). Half of study patients (*n =* 295) presented with TB symptoms, whereas only 13 (4%) standard arm and 7 (2%) intervention arm patients tested positive for TB disease. Among 140 intervention arm patients with TB symptoms, 72% were eligible for same-day initiation. Initiation was higher in the intervention (*n =* 296) versus standard arm (*n =* 297) by 7 days (91% versus 68%; risk difference [RD] 23% [95% confidence interval (CI) 17%–29%]) and 28 days (94% versus 82%; RD 12% [7%–17%]) after enrollment. In total, 87% of intervention and 38% of standard arm patients initiated on the same day. By 8 months after study enrollment, 74% (220/296) of intervention and 59% (175/297) of standard arm patients had both initiated ART in ≤28 days and been retained in care (RD 15% [7%–23%]). Among the 41% of participants with viral load results available, suppression was 90% in the standard arm and 92% in the intervention arm among patients initiated in ≤28 days. No ART-associated adverse events were reported after initiation; two intervention and four standard arm patients were reported to have died during passive follow-up. Limitations of the study included limited geographic generalizability, exclusion of patients too sick to consent, fluctuations in procedures in the standard arm over the course of the study, high fidelity to the trial protocol by study staff, and the possibility of overestimating loss to follow-up due to data constraints.

**Conclusions:**

More than 85% of patients presenting for HIV testing or care, including those newly diagnosed, were eligible and ready for same-day initiation under the SLATE II algorithm. The algorithm increased initiation within 7 days without appearing to compromise retention and viral suppression at 8 months, offering a practical and acceptable approach that can be widely and immediately utilized by existing providers.

**Trial registration:**

Clinicaltrials.gov NCT03315013, registered 19 October 2017. First participant enrolled 14 March 2018.

## Abstract 2

Mhairi Maskew and co-workers evaluate an algorithm for rapid HIV treatment initiation in settings with high tuberculosis symptom prevalence.

## Author summary

**Why was this study done?:**

- Since 2017, health authorities have recommended that treatment for HIV (antiretroviral therapy [ART]) should be started rapidly after a person tests positive for HIV, either on the same day as diagnosis or within 7 days, yet best practices for this process are not yet well described, particularly for those presenting with symptoms of tuberculosis (TB).
- A previous study, Simplified Algorithm for Treatment Eligibility (SLATE) I trial, of a simple procedure for determining whether a patient is clinically eligible and ready for same-day treatment initiation found that the main factor causing delays in treatment was symptoms of TB, which caused patients to have to wait for a TB test before they could start HIV treatment; half of all patients enrolled in South Africa faced delays for this or other reasons.
- SLATE I was feasible and acceptable and led to an increase in treatment uptake, but results pointed to improvements for a revised version of the procedure, or algorithm, that could allow more patients to start ART on the same day.

**What did the researchers do and find?:**

- We conducted the SLATE II study, which allowed patients with mild (nonserious) TB symptoms to start ART on the same day rather than waiting for a TB test.
- We individually randomized 593 patients coming to three public-sector clinics in South Africa for an HIV test or pretreatment care to be offered either same-day ART initiation under the SLATE II algorithm or regular (standard-of-care) procedures.
- We found that treatment initiation within 1 week increased by 23%, and the proportion of patients initiating and staying in care after 8 months increased by 15% in the intervention group; 86% of patients in the intervention group were eligible to start ART on the same day.
- Only about 1 out of 10 patients had serious TB symptoms or other reasons for not starting ART that day; none of the patients who had mild TB symptoms and started ART on the same day reported any serious TB-related illness after ART initiation.

**What do these findings mean?:**

- Same-day ART initiation using a simple algorithm is safe and feasible, can be done by the same nurses who manage ART initiation now, and improves uptake of and retention on ART.
- A structured procedure like SLATE makes it possible for patients with mild symptoms of TB to start ART without waiting for a TB test.
- In the study, nurses followed SLATE II guidelines exactly, so results might be different if the SLATE II approach is scaled up outside a study.

## Introduction

In its 2017 revision of the global guidelines for HIV care and treatment, the World Health Organization (WHO) called for rapid or same-day initiation of antiretroviral therapy (ART) for eligible patients testing positive for HIV [1], with the goal of reducing losses of treatment-eligible patients from care before they receive their first dose of antiretroviral (ARV) medications [2–4]. Several trials and observational studies have reported the benefits of same-day initiation, including higher rates of treatment initiation, shorter intervals between diagnosis and treatment, and ultimately larger proportions of patients achieving viral suppression among all those eligible for treatment [5–10]. In most previous studies, however, a wide range of conditions, including any symptoms of tuberculosis (TB), made patients ineligible for same-day initiation, either by excluding them from study enrollment or delaying ART initiation within the study [7,11]. The large number of patients who present at clinics with mild symptoms associated with TB, such as a cough or fever, or who are otherwise ineligible for the studies are thus generally not reflected in same-day initiation studies, and they do not qualify for same-day initiation under WHO or national guidelines.

Our previous Simplified Algorithm for Treatment Eligibility (SLATE) I study evaluated a simple, accelerated algorithm for ART initiation that required no point-of-care laboratory testing and could be administered in its entirety by nurses, who provide most primary care in South Africa [11]. One of the most striking findings of the study was the large proportion of patients who were ineligible for same-day initiation under the SLATE algorithm and were referred for additional services rather than started on ART immediately. In South Africa, fully half of the patients assigned to the intervention arm and evaluated for immediate treatment eligibility under the SLATE I algorithm screened out of same-day initiation, with two-thirds of these being due to symptoms of TB [11]. Although some patients had multiple symptoms or severe illness, most reported only one symptom of short duration. Despite the frequency of TB symptoms observed, the vast majority of those with TB symptoms (91%) tested negative for active TB disease [12]. These results, as well as findings from the TEMPRANO trial [13], the CASCADE trial [10], and others [14], demonstrate the benefits of early ART among those with TB symptoms and suggest that rapid ART initiation among patients later found to have TB disease and treated for TB does not appear to be of serious concern.

Building on our findings in SLATE I, we developed the SLATE II trial [15] to help meet the need [16] for an improved approach to optimize treatment uptake and early outcomes, particularly in settings with high prevalence of TB symptoms among those presenting for ART. WHO’s 2017 recommendation [1], and adaptations of it in South Africa’s [17] and other countries’ national guidelines, allows or encourages same-day initiation but often provides only general [18] or restrictive [19] guidance on who is eligible. In South Africa, same-day initiation was allowed for patients who were “clinically ready and willing to commit,” without further elucidation of these criteria [17]. SLATE II was an individually randomized evaluation of a revised clinical algorithm that allowed same-day initiation for patients with mild TB symptoms and other less serious reasons for delay and tested a structured set of steps for implementing same-day initiation. We report the SLATE II study’s primary outcomes, ART initiation within ≤7 days of study enrollment and retention in care 8 months after study enrollment, along with several secondary outcomes, including initiation within 0 and 28 days and viral suppression after study enrollment.

## Methods

### Study design and overview

**Fig 1:**
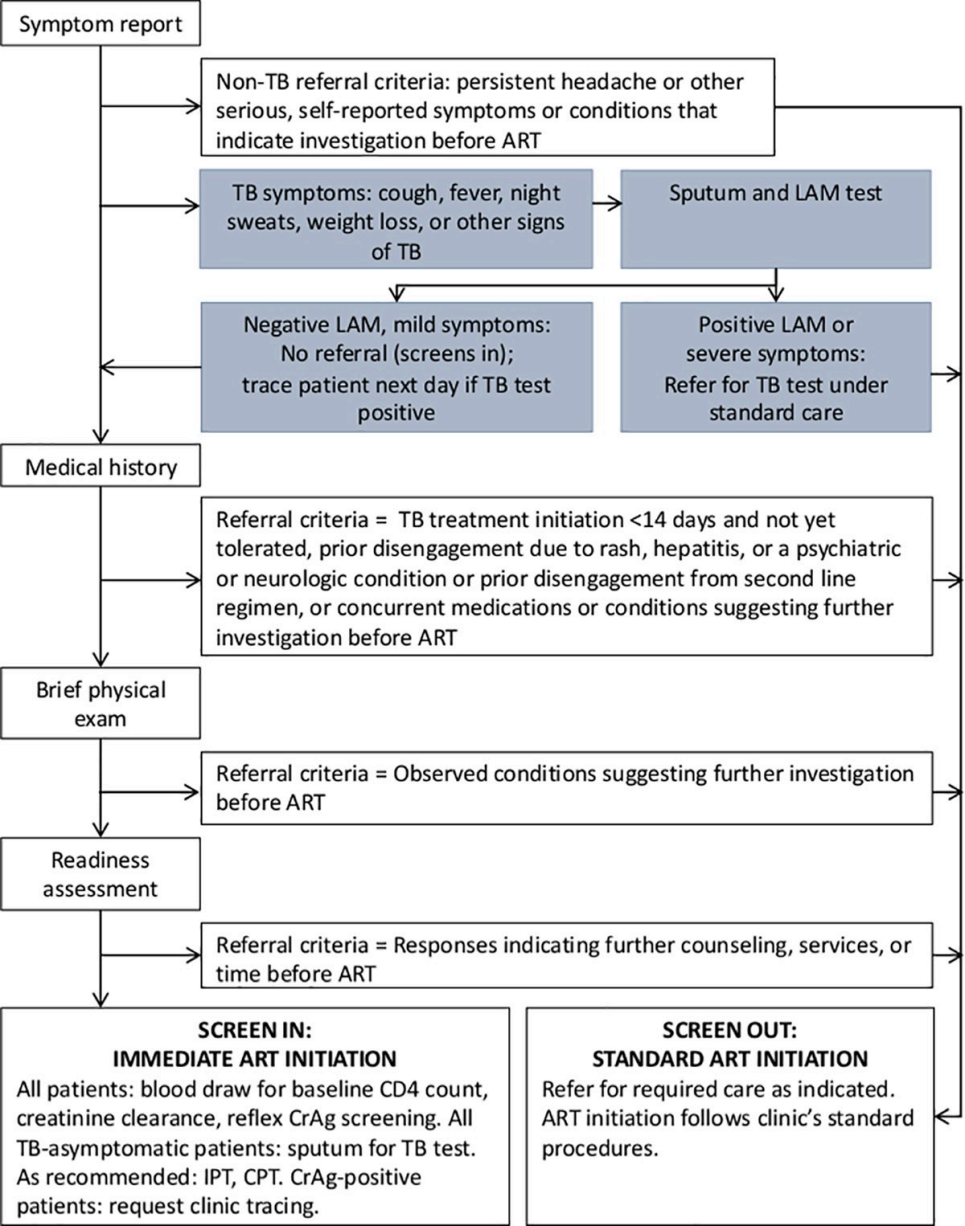
SLATE II algorithm. ART, antiretroviral therapy; CPT, cotrimoxazole preventive therapy; CrAg, cryptococcal antigen; IPT, isoniazid preventive therapy; LAM, lipoarabinomannan antigen of mycobacteria; SLATE, Simplified Algorithm for Treatment Eligibility; TB, tuberculosis.

The SLATE II study, an individually randomized, nonblinded, parallel-group pragmatic evaluation, assessed the effect of the revised SLATE algorithm on ART initiation and retention in care after study enrollment. The algorithm, previously reported in [15] and illustrated in Fig 1, consisted of four screening tools, each evaluating an area of eligibility required for same-day ART initiation: (1) symptom report, (2) medical history, (3) brief physical examination, and (4) patient readiness assessment. Patients randomized to the intervention arm and deemed eligible on all four screening tools were offered initiation of ART on the same day of study enrollment, whereas those who were found to be ineligible on any one of the screens were referred back to routine care at the study site clinic for further management or investigation prior to ART initiation. Differences between the SLATE I and SLATE II algorithms are detailed in S1 Table.

### Study setting

The study was conducted at three high-volume, public-sector primary care clinics based in both urban formal and informal settings in or near Johannesburg, South Africa. During the study period, ART was provided free of charge in the public sector and conformed to WHO guidelines for provision of ART. Study procedures were conducted either in interview rooms allocated to the study team or mobile trailer units placed outside the study site for the duration of the study. The study teams at each site consisted of two study assistants and one nurse practitioner. The study assistants were trained interviewers with data capturing experience. They conducted the recruitment, enrollment and consent, questionnaire, and data capturing of study information onto mobile tablet devices for all patients. The nurse practitioners were all qualified primary healthcare nurses able to dispense ART. They implemented all clinical aspects of the study visit for the intervention arm patients, including the SLATE II algorithm screen. All study team members received both human subject ethics certification and study protocol–specific training.

### Study population and enrollment

The study enrolled all nonpregnant, HIV-infected adults not yet on ART. Inclusion criteria were age ≥18 years, a confirmed HIV-positive test result at any time (may have been diagnosed previously), self-report that patient is not currently on ART and has not been prescribed ART in the past 3 months, and presentation at the study clinic for any reason that led to referral for HIV testing or care. Patients were excluded if they were pregnant (pregnancy was an exclusion criterion because treatment guidelines for pregnant women differ from those for nonpregnant adults; most pregnant women are diagnosed with HIV and initiated on ART in antenatal clinics, not general adult HIV clinics); did not intend to return to this clinic for further HIV care in the coming year; refused to be traced by phone or in person for follow-up care if test results received after the enrollment visit indicated that further care was needed; were not physically, mentally, or emotionally able to participate in the study in the opinion of the investigators or study staff; were not willing or able to provide written informed consent to participate in the study; or had previously enrolled in the same study or the SLATE I study. Eligible patients included both those newly diagnosed on the day of study enrollment and those who had already visited the study clinic (or another) for HIV-related care, provided that they had not yet started ART.

Potentially eligible study participants were informed about the study at visit registration or after a positive HIV test at one of the participating study sites and screened by a study assistant to confirm study eligibility. Eligible patients who were willing and able provided written informed consent to participate. Those who refused consent were referred back to the clinic queue to complete their routine visit.

### Study procedures—All patients

**Fig 2:**
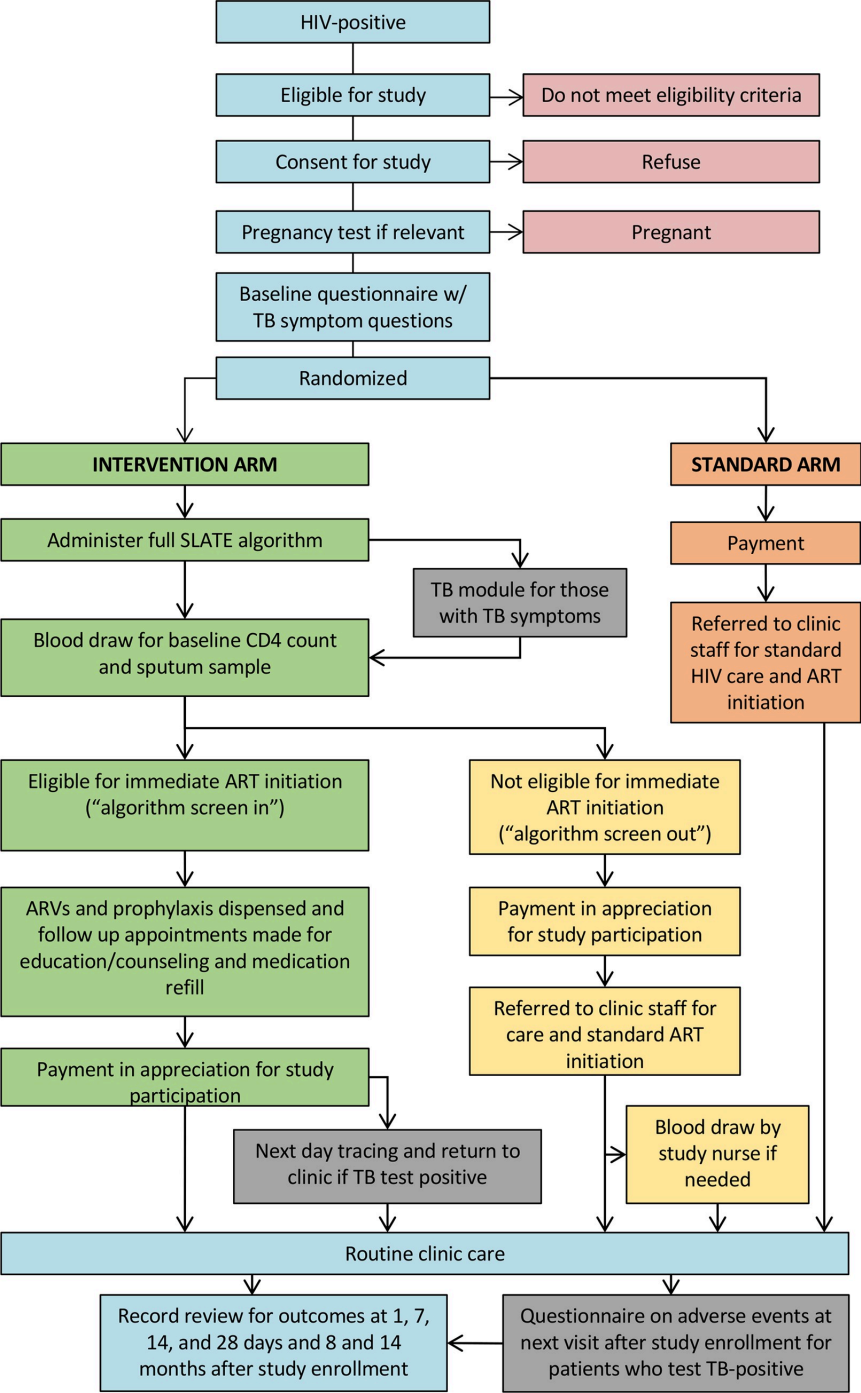
SLATE II trial procedures. ART, antiretroviral therapy; SLATE, Simplified Algorithm for Treatment Eligibility; TB, tuberculosis.

Study procedures are illustrated in Fig 2. After consent and enrollment, all female study participants performed a rapid urine pregnancy test to identify women unaware of being pregnant at the time of study enrollment. After exclusion of pregnant women, all study participants completed an interviewer-administered questionnaire exploring patients’ demographic characteristics, HIV history, knowledge and treatment preferences, employment and primary activities, and visit costs. On completion of the questionnaire, study participants were then randomized 1:1 to either the intervention arm (SLATE algorithm) or standard arm (routine clinic procedures). The randomization sequence was generated by a computerized random number generator, and block randomization in blocks of six was implemented by the study PI and assigned to study sites in complete blocks of six to maintain the integrity of the randomization sequence. Study allocations were numbered and stored in sealed envelopes at the study site and were used sequentially. No blinding of patients or study staff was possible because each allocation involved different procedures.

During the study period, treatment-naïve HIV patients were initiated onto the standard first-line ARV regimen of tenofovir, emtricitabine, and efavirenz, dispensed in a combined once-daily tablet. All study participants received a payment equivalent in value to US$12 to thank them for participating and compensate them for their time.

### Study procedures—Standard arm

After randomization, standard arm patients were accompanied back to the appropriate clinic queue to continue their treatment under routine care settings. In general, national guidelines for ART initiation [20] were followed for standard arm patients, including the possibility of same-day initiation, which was encouraged in national guidelines as of 2017 for patients considered clinically eligible and willing [17]. Procedures for ART workup at all sites included a preinitiation creatinine clearance test and a CD4 count with reflexive cryptococcal antigen (CrAg) testing for CD4 counts ≤100 cells/mm^3^. One site performed RPR (syphilis) testing at initiation, whereas patients initiating at another site received hemoglobin, alanine aminotransferase, and hepatitis B virus tests. The results of these tests were generally available ≥1 day later, not soon enough to influence same-day initiation eligibility. At two of the sites, patients presenting with two or more TB symptoms were asked for a sputum sample, which was tested at a centralized laboratory using Xpert MTB/RIF. The third site routinely offered TB testing to all HIV-positive patients regardless of symptom history. TB test results were typically available the next working day, though the sites varied in how promptly they accessed the results. Chest X-ray facilities were available at all study sites for patients when it was clinically indicated by the treating clinician. Study procedures for standard arm patients (screening, consent, questionnaire) required approximately 40 minutes per patient.

### Study procedures—Intervention arm

After being informed of their study arm allocation, the study nurse administered the four screening components of the SLATE algorithm to patients randomized to the intervention arm. Each screen is intended to identify specific reasons that a patient might not be eligible, including current symptoms of opportunistic infections, previous experiences or behaviors that indicate a need for additional treatment readiness or adherence support, and the patient’s own concerns about starting treatment. The algorithm captured all components of treatment eligibility assessment currently used in routine care in South Africa.

As shown in Fig 1 and S1 Table, one of the primary differences between the SLATE I and SLATE II algorithm is how intervention arm patients reporting TB symptoms at study enrollment were managed. For SLATE I, all patients reporting one or more TB symptoms of any severity or duration were considered ineligible for same-day ART and were referred back to the clinic to follow standard procedures. Under SLATE II, patients with TB symptoms were clinically evaluated by the study nurse, who conducted a urine point-of-care lipoarabinomannan antigen of mycobacteria (LAM) test (Determine TB LAM Ag, Abbott, Waltham, MA, USA). Those with mild symptoms (for example, reporting one or two symptoms only of less than 2 weeks’ duration), no examination findings of concern, and a negative LAM test were offered immediate ART. Those with more serious symptoms (e.g., productive cough of more than 2 weeks’ duration or presence of three or more symptoms) and/or a positive LAM test were referred back to standard care for TB testing and management. All intervention arm patients (symptomatic and asymptomatic) were asked for a sputum sample for Xpert testing, and positives were contacted on the next working day. Study nurses applied their clinical judgment as they administered all of the screens to avoid unnecessarily delaying those who are eligible for immediate initiation and to ensure appropriate additional care for those who need it. Upon completion of the four SLATE screens, nurses dispensed ART directly to those patients eligible for same-day initiation and provided referral letters for those patients requiring additional investigation or services prior to initiation of ART. The study procedures are outlined in detail in the previously published protocol [15].

### Study outcomes

The study had two primary outcomes: (1) initiation of ART within 7 days of study enrollment and (2) initiation of ART within 1 month (28 days) and retention on ART 8 months after study enrollment. Seven days is WHO’s definition of “rapid” initiation [1]. Retention is measured as having an observed clinic visit in either the patient’s paper record or the site’s electronic patient register between 5 and 8 months after study enrollment. Eight months was selected to allow up to 1 month to initiate ART, 6 months of follow-up after treatment initiation, and up to 1 month to return for the 6-month routine clinic visit. Electronic and hard copy clinic records were searched for confirmation of death, transfer to another site, or loss from care. Patients with no evidence of a clinic visit or laboratory test between 5 and 8 months after study enrollment were assumed lost to follow-up. We also report the following secondary outcomes: time to ART initiation; proportion initiating ART within 0 (same-day), 14, and 28 days of study enrollment; suppression of viral load (to <400 copies/mL) by 8 months after study enrollment; proportions screening out of same-day initiation and reasons for ineligibility; patient preference on timing of ART initiation; frequency of adverse events; and proportion of TB symptomatic patients testing positive for TB. Viral suppression and nonsuppression are reported based on known test results; patients without viral load tests are reported in a separate group. Protocol-defined secondary outcomes related to costs and health system outcomes will be reported in a separate manuscript, as will outcomes after 14 months’ follow-up.

### Data collection

Data were collected from two sources. First, at the study enrollment visit, a case report form (CRF) was completed, with eligibility and questionnaire data for all participants and SLATE algorithm data for intervention arm participants. CRF data were entered by study staff onto tablet computers programmed for data collection using REDCap Mobile [21]. Second, all follow-up data were sourced from routinely collected medical records, primarily TIER.Net [22], South Africa’s national HIV monitoring system. Data from TIER.Net were supplemented with routinely generated clinical record data from patient records in electronic and paper format, including laboratory records from the National Health Laboratory System (NHLS) and clinic registers as needed.

### Data analysis

We commenced with a simple comparison of the two study arms with respect to baseline predictors of outcomes to look for any imbalances in these characteristics of the enrolled sample. We report simple proportions and medians with interquartile ranges (IQRs) stratified by study arm.

Next, we conducted a crude analysis comparing the proportion of patients achieving each dichotomous outcome by study arm. We estimated crude risk ratios and crude risk differences (RDs) and their corresponding 95% confidence intervals (CI) for each of the specific outcomes as described. For all outcomes, the analytic approach was by intention to treat: subjects were analyzed according to the intervention they were supposed to receive, whether or not they adhere to the defined intervention. This included patients randomized to the intervention arm who screened out of immediate ART initiation by the SLATE II algorithm; they remained in the intervention arm for data analysis.

Finally, as prespecified, we looked for effect modification by predictors of each outcome: age, sex, site, CD4 count at enrollment, reason for clinic visit, and presence of one or more TB symptoms at study enrollment. We used a simple stratification of the primary analysis by the potential modifier and report crude RDs and risk ratios and their corresponding 95% CIs. We then fit generalized linear models to assess any relative excess due to interaction (RERI) and present relative risks (RRs) with associated 95% CIs.

### Sample size

Based on the results of the SLATE I study, we estimated that 60% of treatment-eligible patients would be initiated on ART within 7 days in the standard arm, and we considered an increase to 75% to be programmatically important. Using an α of 0.05, power of 90%, 1:1 randomization, and an uncorrected Fisher’s exact test, this required a minimum sample size of 200 patients per group. We increased our total target sample size to 600 to allow for exclusion of pregnant patients after consent and for effect measure modification, including an analysis of outcomes by study site.

### Ethics

The study was approved by both the Human Research Ethics Committee of the University of the Witwatersrand (Medical) (171011) and the institutional review board of Boston University Medical Campus (H-37010) and is registered with clinicaltrials.gov (NCT03315013).

**Fig 3:**
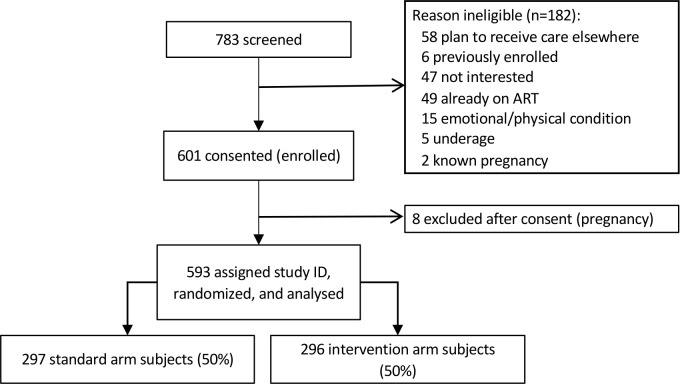
CONSORT flow diagram for the SLATE II randomized trial of same-day treatment initiation. ART, antiretroviral therapy; ID, identifier; SLATE, Simplified Algorithm for Treatment Eligibility; CONSORT, Consolidated Standards of Reporting Trials.

This study is reported per CONSORT guidelines (Fig 3 and CONSORT checklist, S1 Text).

## Results

Of the 783 patients approached to participate in the study across the three sites, we enrolled a total of 601 (77%) between 14 March and 18 September 2018 (Fig 3). Among the 182 patients not eligible for study enrollment; 58 (32%) planned to receive care at another facility; 49 (27%) were already receiving ART; 47 (26%) were not interested in participating; 15 (8%) were physically or emotionally unable to participate; and the rest were <18 years of age, known to be pregnant, or previously enrolled in the study. After enrollment, a further eight women had a positive pregnancy test and were excluded, leaving a total of 593 study participants, who were randomized to either the standard arm (*n =* 297, 50%) or the intervention arm (*n =* 296, 50%) and were analyzed by original assigned groups. Patients were followed up passively through record review until 31August 2019. This allowed all participants a minimum of 8 months of potential follow-up time after study enrollment and an additional 3 months for records to be captured in clinic electronic registers and databases.

### Characteristics of patients at study enrollment

Self-reported demographic, clinical, and care-seeking characteristics of patients at study enrollment are summarized in Table 1. The study population was predominantly female (63%), with a median age of 35 years (IQR 29–43) and median CD4 count of 293 cells/mm^3^ (IQR 133–487). More than half (56%) reported being single, and nearly half (49%) were unemployed and seeking work at study enrollment. Fewer than half of patients (45%) reported having tested for HIV previously; most patients (68%) were visiting the study site clinic for the first time. Nearly all patients (98%) said that they would want to start ART on the day of study enrollment if they could. No important imbalances with respect to the characteristics of the enrolled sample were noted. Additional details about the study cohort have been reported elsewhere [23].

**Table 1:** Characteristics of SLATE II study patients at enrollment by arm.

Characteristic at study enrollment	Standard arm (*n =* 297)	Intervention arm (*n =* 296)
Site		
Site 1 (City of Johannesburg)	102 (34%)	102 (34%)
Site 2 (Ekurhuleni)	107 (36%)	105 (36%)
Site 3 (City of Johannesburg)	88 (30%)	89 (30%)
Age (median [IQR])	35 (30–44)	35 (29–41)
Gender (female)	184 (62%)	189 (64%)
CD4 count (cells/mm^3^)		
Median (IQR)	293 (127–514)	298 (137–482)
≤100	57 (19%)	51 (17%)
101–200	50 (17%)	55 (19%)
201–350	58 (20%)	60 (20%)
350–500	41 (14%)	61 (21%)
>500	77 (26%)	64 (22%)
Missing	14 (4%)	5 (1%)
Current residence		
Rural home or village	1 (0%)	0 (0%)
City (urban)	29 (10%)	31 (10%)
Township (periurban)	267 (90%)	265 (90%)
Current house is primary residence (yes)	131 (44%)	128 (43%)
Other persons in house (median [IQR])	2 (1–3)	1 (1–3)
Marital status		
Married or living with long-term partner	107 (36%)	94 (32%)
Single	158 (53%)	172 (58%)
Divorced, separated, or widowed	32 (11%)	30 (10%)
Activity when well		
Formal work	45 (15%)	69 (23%)
Informal work	82 (28%)	70 (24%)
Unemployed, seeking work	144 (49%)	138 (47%)
Other	16 (5%)	8 (2%)
Missing	10 (3%)	11 (4%)
Transport mode		
Walking	158 (53%)	161 (54%)
Minibus taxi or bus	120 (40%)	110 (37%)
Motorbike	1 (1%)	0 (0%)
Private car	18 (6%)	25 (9%)
Had to pay for travel (yes)	142 (48%)	136 (46%)
Travel time (minutes one-way) median (IQR)	15 (10–25)	15 (10–30)
Previously came for care at this clinic (yes)	95 (32%)	94 (32%)
Main reason for today’s clinic visit		
HIV test	110 (37%)	108 (36%)
Other HIV care	127 (43%)	141 (48%)
Other reason	60 (20%)	47 (16%)
Previously tested for HIV? (yes)	135 (45%)	133 (45%)
If tested, status disclosed to anyone else? (yes)	92 (68%)	111 (83%)
If tested, year first tested positive		
During 2018	56 (41%)	53 (40%)
During 2017	22 (16%)	21 (16%)
Prior to 2017	57 (42%)	59 (44%)
Expected to start ART today? (yes)	171 (58%)	183 (62%)
Have enough HIV information to start ART today?		
I already know enough	197 (66%)	201 (68%)
I would like a little more information	73 (25%)	65 (22%)
I would like a lot more information	27 (9%)	30 (10%)
When would patient want to start ART?		
Today	295 (99%)	289 (98%)
Within the next week, but not today	2 (1%)	6 (2%)
Within the next month, but not this week	0 (0%)	1 (0%)

Abbreviations: ART, antiretroviral therapy; IQR, interquartile range; SLATE, Simplified Algorithm for Treatment Eligibility

### ART initiation within 7 days and timing of initiation

Table 2 reports treatment initiation outcomes. In the standard arm, 202/297 patients (68%) initiated ART within 7 days of study enrollment. In the intervention arm, this figure was 270/296 patients (91%), for an absolute RD of 23% (95% CI 17%–29%). We noted the proportion of patients initiating ART was higher at every time point assessed. Even 90 days after enrollment, 44 (15%) patients in the standard arm had not yet started treatment, compared with 16 (5%) in the intervention arm. Most patients in the intervention arm (87%) started ART on the day of study enrollment (same-day initiation); 38% of patients in the standard arm did so.

**Table 2:** Initiation outcomes by study arm.

Outcome	Standard arm (*n =* 297)	Intervention arm (*n =* 296)	Crude risk difference (95% CI)^†^	Crude relative risk (95% CI)^†^
**Initiation ≤7 days**				
Initiated ≤7 days	202 (68%)	270 (91%)	23% (17%–29%)	1.34 (1.23–1.46)
Record found, did not initiate ≤7 days	77 (26%)	21 (7%)	−19% (−25% to −13%)	0.27 (0.17–0.43)
No record found, assumed did not initiate ≤7 days	18 (6%)	5 (2%)	−4% (−7% to −1%)	0.28 (0.10–0.74)
**Time to initiation**				
Initiated in 0 days (same day)	114 (38%)	257 (87%)	49% (42%–55%)	2.26 (1.95–2.63)
Initiated within 14 days	228 (77%)	274 (93%)	16% (10%–21%)	1.21 (1.12–1.29)
Initiated within 28 days	243 (82%)	277 (94%)	12% (7%–17%)	1.14 (1.08–1.22)
No record of initiation ≤28 days	54 (18%)	19 (6%)	−12% (−17% to −7%)	0.35 (0.21–0.58)

^†^Reference group: standard arm.

Abbreviation: CI, confidence interval

### Algorithm results

Of the 296 patients enrolled in the intervention arm, 255 (86%) were eligible for same-day initiation under the SLATE II algorithm. Of the 41 who were not eligible for same-day initiation, 2 (5%) presented with physical conditions requiring further investigation or management prior to ART start, and 39 (95%) presented with TB symptoms serious enough to warrant delay of ART initiation. Of these, 11 presented with only TB symptoms, whereas the remaining 28 had both TB symptoms and one or more other criteria for delaying ART initiation. These included persistent headache (*n =* 8), concerning clinical condition unrelated to TB (*n =* 6), patient not ready to start (*n =* 5), medical history (*n =* 2), or a combination of two of these reasons in addition to TB symptoms (*n =* 7). Two of the 41 patients who screened out of same-day ART in the intervention arm were initiated on the day of enrollment by clinic staff following study referral, resulting in a total of 257 intervention arm patients who initiated ART on the day of study enrollment.

A total of 213 (36%) patients were enrolled with a CD4 count <200 cells/mm^3^ (107 [36%] in the standard arm, 106 [36%] in the intervention arm). Among the 106 patients in the intervention arm with CD4 counts <200, 78% (*n =* 83) initiated ART on the day of study enrollment, and 83% (*n =* 88) initiated within 7 days. In the standard arm, 62% (*n =* 66) of patients presenting with a CD4 count <200 initiated within 7 days (S2 Table).

Among intervention arm patients who were asked about prior ART use, 33 (11%) stated that they had previously been on ART, and prior initiation was a median of 4 years (IQR 2–7 years) before study enrollment.

### TB at study enrollment

At study enrollment, approximately half of standard (52%, *n =* 155) and intervention (47%, *n =* 140) arm patients presented with one or more TB symptoms. Details of the intervention arm patients are reported elsewhere [23]. Overall, 392 (66%) of study patients were willing and able to produce a sputum sample and were tested for TB at study enrollment, including 163 (55%) standard arm patients and 229 (77%) in the intervention arm. In total, 3% (*n =* 20) of study patients tested positive for TB at enrollment, comprising 13 (4%) standard arm and 7 (2%) intervention arm patients. All but one of the intervention arm patients (6/7) who tested positive for TB were symptomatic at enrollment.

According to the SLATE II algorithm, 39 (28%) of the 140 patients presenting with TB symptoms were not eligible for same-day initiation. In total, 74% of intervention arm patients presenting with TB symptoms were initiated on the day of study enrollment, and 82% had been initiated within 7 days. Of the seven confirmed TB cases in the intervention arm, two were initiated on the same day under the SLATE II algorithm, one was initiated on the day of study enrollment by clinic staff after being referred by the study for further investigation, two were initiated within 28 days of study enrollment, and the remaining two had no records of initiating ART.

In the standard arm, only 28% of patients presenting with TB symptoms were initiated on the day of study enrollment, and 62% had been initiated within 7 days. Of the 13 confirmed TB cases, none were initiated on the same day; three and a further five were initiated within 7 and 28 days of study enrollment, respectively; and the remaining five had no records of initiating ART.

As reported elsewhere [12], among the 140 symptomatic patients in the intervention arm tested with LAM, two had positive results. One of these also tested positive with Xpert MTB/RIF and was initiated on TB treatment, whereas the other tested negative with Xpert and was not considered to have TB. No serious postinitiation TB-related adverse events were reported during the 8 months.

### TB diagnosis after study enrollment

We traced TB records for study participants up to 8 months after study enrollment. In total, 16 study participants (nine standard and seven intervention arm patients) were investigated for TB by the study clinics. Of these, five were confirmed TB positive (two within 1 week of study enrollment and three between 7 and 8 weeks after enrollment). Two started TB treatment at the study sites, two were transferred to other facilities for TB treatment, and one had no record of receiving TB treatment.

### Retention and viral suppression

Our second primary outcome was a composite of ART initiation within 28 days and retention in care at 8 months after study enrollment, as indicated by a clinic visit in months 5–8 (Table 3). In total, 59% (175/297) of standard arm patients achieved this outcome, compared with 74% (220/296) of intervention arm patients, for an RD of 15% (8%–23%). Roughly one-quarter of all patients who did initiate ART within 28 days were not retained at 8 months, with little apparent (−3% [95% CI −10% to 3%]) difference in confirmed or assumed loss to follow-up between the arms. Among the 20% of standard arm patients and 17% of intervention arm patients confirmed or assumed to have been lost to follow-up within 8 months, 10 and 15 patients, respectively, never returned to the site after starting ART, whereas the rest made one or more postinitiation visits.

**Table 3:** Retention and viral suppression after initiation ≤28 days by study arm.

Outcome	Standard arm (*n =* 297)	Intervention arm (*n =* 296)	Crude risk difference (95% CI)^†^	Crude relative risk (95% CI)^†^
**Retained in care by 8 months**				
Initiated ART ≤28 days and retained in care 8 months after study enrollment*	175 (59%)	220 (74%)	15% (8%–23%)	1.26 (1.12–1.42)
Initiated ART ≤28 days, not retained 8 months after study enrollment	68 (23%)	57 (19%)	−4% (−10% to 3%)	0.84 (0.62–1.15)
Not retained—died	4 (2%)	2 (1%)	−1% (−2% to 1%)	0.50 (0.09–2.72)
Not retained—transferred	5 (1%)	5 (2%)	1% (−1% to 3%)	1.67 (0.40–6.93)
Not retained—confirmed or assumed LTFU	59 (20%)	50 (17%)	−3% (−10% to 3%)	0.85 (0.60–1.20)
No record of initiation ≤28 days	54 (18%)	19 (7%)	−11% (−17% to −7%)	0.35 (0.21–0.58)
**Viral suppression by 8 months**				
Initiated ART ≤28 days and known to be virally suppressed ≤8 months**	94 (32%)	130 (44%)	12% (5%–20%)	1.39 (1.12–1.71)
Initiated ART ≤28 days and not known to be virally suppressed ≤8 months	81 (28%)	90 (30%)	2% (−5% to 10%)	1.10 (0.86–1.42)
Known unsuppressed VL result	11 (4%)	11 (4%)	0% (−3% to 3%)	1.00 (0.44–2.28)
VL result not traced	70 (24%)	79 (27%)	3% (−4% to 10%)	1.12 (0.85–1.47)
Initiated ART ≤28 days but not retained in care	68 (23%)	57 (19%)	−4% (−5% to -9%)	0.88 (0.67–1.16)
No record of initiation ≤28 days	54 (18%)	19 (7%)	−11% (−17% to −7%)	0.35 (0.21–0.58)

*Retained = initiated ART ≤28 days and had an observed clinic visit or VL result 5–8 months after study enrollment.

^†^Reference group: standard arm.

**VL suppression = initiated ART ≤28 days and suppressed VL (<400 copies/mL) observed 5–8 months after study enrollment.

Abbreviations: ART, antiretroviral therapy; CI, confidence interval; LTFU, lost to follow-up; VL, viral load

Fewer than half of patients in both arms had viral load test results reported in their files by 8 months after study enrollment. Among those with results reported, suppression was 90% (94/105) in the standard arm and 92% (130/141) in the intervention arm. Both arms had high rates of missing viral load tests (patients retained in care but without viral load results reported): 24% in the standard arm and 27% in the intervention arm. Expanding the window for viral load testing to include a range of 2–8 months after study enrollment did not meaningfully alter the difference between the arms in terms of proportions initiated ≤28 days and achieving viral suppression (S3 Table).

As Table 3 indicates, six patients who initiated ART in ≤28 days are known to have died during study follow-up. One additional standard arm patient died without having initiated ART (S4 Table). Investigators concluded that none of these deaths were related to study procedures. In addition, one intervention arm patient who was eligible for same-day initiation and did start ART on the same day was found to have an elevated serum creatinine. This patient was contacted and advised to return to the clinic for regimen modification. (This patient was retained in care at 8 months, achieving the study’s second primary outcome.)

### Absolute effect modification by key variables

Secondary outcomes included an analysis of effect modification by key variables. As shown in S2 Table, the most important potential modifier was site, with site 1 accounting for a disproportionate share of overall impact. For initiation within 7 days, the RD and RR at this site were 36% (25%–48%) and 1.69 (1.39–2.05), respectively. At the other two sites, initiation in ≤7 days showed more modest, though still potentially important, RDs (RD 14% [95% CI 3%–24%] for site 3 and RD 18% [95% CI 9%–28%] for site 2) and RRs (RR 1.18 [95% CI 1.04–1.33] for site 3 and RR 1.25 [95% CI 1.10–1.42] for site 2). The large effect at site 1 may partly be explained by the relatively low rate of initiation in ≤7 days in the standard arm at this site. For initiation within 28 days and retention at 8 months, the RD and RR for site 1 were 19% (5%–32%) and 1.37 (1.09–1.73), respectively. Again, differences in retention for the other sites were smaller; RDs between arms of 12% (95% CI 0%–23%) for site 2 and 16% (95% CI 2%–30%) for site 3. Although these comparisons were prespecified, the study was not powered for effect modification analysis. These results should thus be considered hypothesis generating. We did not find important effect measure modification for any of the other variables investigated (S2 Table).

## Discussion

The SLATE II study successfully initiated onto ART more than 90% of nonpregnant adult patients who presented at primary healthcare clinics within 7 days and demonstrated that nearly 90% of patients are eligible for same-day ART initiation according to the SLATE II algorithm. At 8 months later, patients offered the SLATE II intervention were 26% more likely to have initiated within 28 days and then subsequently be retained in care compared with those in standard care. The intervention thus achieved improvements in both primary outcomes compared with standard care without generating any difference in known postinitiation adverse event rates.

As mentioned above, the SLATE II algorithm was designed to address the shortcomings of the SLATE I algorithm, for which eligibility criteria for same-day initiation were found to be too conservative. The two studies are compared side-by-side in S5 Table. The proportion of patients initiating ART on the day of study enrollment increased in both arms between SLATE I and SLATE II and indicates that initiation times appear to be decreasing in standard care, though same-day initiation achieved its highest level (87%) using the SLATE II algorithm. Initiation within 28 days followed a similar pattern, reaching a high of 94% in SLATE II, compared with 78% in SLATE I. Initiation and subsequent retention at 8 months after study enrollment, which hovered near 50% in both arms in SLATE I and in the standard arm in SLATE II, rose to 70% in the intervention arm of SLATE II.

Concerns that same-day initiation promotes loss to follow-up after treatment initiation were not realized in the SLATE II study. The intervention showed sustained benefit in terms of retention and viral suppression among patients who did initiate ART within 28 days. That said, retention was not sufficient in either study arm—74% in the intervention arm and a disappointing 59% in the standard arm—suggesting that other interventions to improve retention after initiation are still needed. Similarly, although viral suppression reached or exceeded 90% in both arms among those who had viral load tests, overall suppression rates—32% and 44%—were also inadequate, highlighting the large proportion of patients for whom viral load test results could not be found. It is not clear whether these missing tests were not done or the results were never returned. We also note that both retention in care and viral suppression are likely to be underestimated by the routine data source used in our study, Tier.Net; actual retention and suppression in both arms may well have been considerably higher than reported [24]. Regardless, same-day initiation, done according to the SLATE II algorithm, did not appear to compromise patient retention or suppression compared with standard-of-care initiation.

In addition to the RapIT and SLATE I trials conducted in South Africa and cited above, several other evaluations of same-day ART initiation have been conducted in recent years. In particular, it is useful to compare our findings with those of a recent observational study of routine ART initiation in two districts in South Africa during the period after the National Department of Health recommended same-day initiation [25]. In that study, Lilian and colleagues found that same-day initiators were more likely to be lost from care 6 months after initiation than those who took longer to start ART. The study did not estimate preinitiation loss to follow-up, however, thus missing the potential benefits of same-day initiation in reducing losses before initiation. Moreover, because patients appear to have opted for either same-day initiation or delayed initiation or had this decision made for them by a clinician, selection bias may in part explain the discrepancy. It is also possible that the SLATE II algorithm itself, combined with the quality of service delivery in our intervention arm, is partially responsible. Lilian and colleagues note that for some patients, same-day initiation may shift attrition from before to after initiation, a premise with which we agree [11,26]. At the same time, the higher initiation rate in our intervention arm, combined with the similar proportions observed as lost from care among those who did start ART within 28 days, suggests that the offer of same-day initiation, as guided by the SLATE II algorithm, prompts patients to start who might otherwise postpone taking action and sends them away with medications in hand, which is what they say they want and has in itself has been shown to improve retention [27]. We thus believe that an optimal strategy will be to offer high-quality same-day initiation, followed by more effective retention support for newly initiated patients.

By design, SLATE II used less conservative criteria for determining eligibility for same-day initiation than have previous clinical trials, including our own. In particular, SLATE II took a different approach to the management of patients with symptoms of TB, allowing patients with short-term and/or mild symptoms and a negative LAM test to initiate ART immediately, rather than awaiting the results of a TB test. This prevented unnecessary delays in ART initiation among the 95% (133/140) of intervention arm patients with one or more TB symptoms who did not have TB, without producing any serious, reported, TB-related adverse events after ART initiation. Testing for TB among asymptomatic patients in the intervention arm of the trial identified only one additional case of TB, suggesting that universal TB testing, regardless of symptoms, may not prove to be a worthwhile intervention in this population. Further research on this question is warranted.

In addition, more than 80% of intervention arm patients presenting with CD4 counts less than 200 cells/mm^3^ initiated ART within 7 days of study enrollment, greater than 20% more than in the standard arm. As WHO guidelines indicate that patients with advanced HIV disease should be prioritized for rapid (≤7 days) initiation of ART, our results suggest that the SLATE II algorithm offers a simple and safe approach to managing initiation in this group.

Generalizability of the impact of interventions to “real-world” settings is often a concern when interpreting results of clinical trials, even those designed to reflect routine conditions, such as SLATE II. In contrast to SLATE I, the SLATE II intervention produced potentially important improvements in both primary outcomes at all three study sites, including site 2, which had quite good performance in the standard arm compared with sites 1 and 3. Although we cannot assume that results from three clinics in a single province are fully generalizable to South Africa, or the region as a whole, the consistency of our findings suggests that many sites may expect similar outcomes with implementation of the SLATE II algorithm. In addition, our results saw high rates of uptake of ART among intervention arm patients regardless of their reason for attending the study site on the day of enrollment (94% among those attending for an HIV test versus 89% among those attending for another reason; S2 Table). We saw similar effects of the SLATE II algorithm on initiation within 7 days by reason for clinic visit (RD 21% [95% CI 12%–30%] among those attending for an HIV test versus RD 25% [95% CI 17%–32%] among those attending for another reason) and on retention at 8 months by reason for clinic visit (RD 13% [95% CI 1%–25%] versus RD 17% [95% CI 7%–26%]). These results suggest that many individuals not actively seeking HIV care are still willing and able to initiate ART when discovered to be infected.

The study had a number of limitations, most of which are mentioned in the previously reported protocol and publication on SLATE I. We have addressed geographic generalizability above. We note that study eligibility criteria excluded patients who were too ill to participate, potentially biasing our sample toward healthier patients. Because this was a pragmatic trial, moreover, with no direct control over the care provided to the standard arm, the quality of services provided to standard arm patients may have fluctuated over the enrollment period and by study site. Due to our reliance on routinely collected data and the absence of unique identification numbers in the South African health system, we could not trace or ascertain the true outcomes of patients who appeared to be lost to follow-up. Some of these patients may have transferred care unofficially to other clinics, and others may have died. To the extent that this occurred, our second primary outcome may overestimate true losses from care. Finally, as we noted for SLATE I, the intervention arm of SLATE II was implemented by trained study staff who achieved near-perfect fidelity to intervention procedures; we might not expect such consistent implementation in routine care settings, and the effect reported may thus not reflect what would be seen in practice.

Throughout sub-Saharan Africa, many countries have introduced the possibility of same-day ART initiation into their national HIV programs. Although it is usual to recommend that same-day initiation be offered only to patients who are “eligible” or “ready,” and there is consensus that those who refuse treatment or require inpatient admission do not meet these criteria, there is little detailed guidance on exactly who should be deemed “eligible” for same-day initiation or how to implement it. With SLATE II, we have shown that a simple but carefully structured algorithm can be used by existing healthcare personnel to distinguish patients who can start ART that day, even if they have mild symptoms of illness, from those who do require additional care prior to initiation. Although we anticipate that each healthcare system—and in fact each clinic or program—will tailor the algorithm to its own needs and priorities, SLATE II offers a starting point that promises to help achieve global goals for ART uptake and retention in care.

## Supporting information

S1 Text CONSORT checklist. CONSORT, Consolidated Standards of Reporting Trials.
(PDF)

S1 Table Differences between the SLATE I and SLATE II algorithms. SLATE, Simplified Algorithm for Treatment Eligibility.
(DOCX)

S2 Table Effect measure modification by key variables. (DOCX)

S3 Table Viral suppression with a window of 2–8 months for test results. (DOCX)

S4 Table Mortality reported during study follow-up. (DOCX)

S5 Table Comparison of SLATE I and SLATE II results. SLATE, Simplified Algorithm for Treatment Eligibility.
(DOCX)

S1 Data SLATE II algorithm instrument. SLATE, Simplified Algorithm for Treatment Eligibility. (PDF)

## Abbreviations

ART: antiretroviral therapy
ARV: antiretroviral
CI: confidence interval
CONSORT: Consolidated Standards of Reporting Trials
CrAg: cryptococcal antigen
CPT: cotrimoxazole preventive therapy
CRF: case report form
IPT: isoniazid preventive therapy
IQR: interquartile range
LAM: lipoarabinomannan antigen of mycobacteria
NHLS: National Health Laboratory System
RD: risk difference
RERI: relative excess due to interaction
RR: relative risk
SLATE: Simplified Algorithm for Treatment Eligibility
TB: tuberculosis
WHO: World Health Organization
