# Supplementary material for: A clinical algorithm for same-day HIV treatment initiation in settings with high TB symptom prevalence in South Africa: The SLATE II individually randomized clinical trial
Source: PLoS Med. 2020 Aug 27;17(8):e1003226. doi: 10.1371/journal.pmed.1003226 (PMC7451542; doi:10.1371/journal.pmed.1003226)
Supplement: S1 Table — SLATE, Simplified Algorithm for Treatment Eligibility. (DOCX) [file pmed.1003226.s002.docx]

### **S1 Table. Differences between the SLATE I and SLATE II algorithms***

| **Screen** | **SLATE I approach** | **SLATE II approach** |
| --- | --- | --- |
| Symptoms | Any symptom of TB (cough, fever, night sweats, weight loss) of any duration or severity led to screening out. | New TB module for patients with any symptoms of TB, including clinical investigation by nurse and LAM test. Patients with mild symptoms and negative LAM were eligible for SDI, while patients with more severe symptoms or positive LAM screened out. |
| Medical history | All previous defaulters screened out. | Previous defaulters eligible for SDI unless the default was due to side effects from the current ARV regimen. |
|  | Any self-report of substance use led to screening out. | No screening out for substance abuse except where patient was ill. |
|  | TB treatment initiated within 14 days led to screening out. | ﻿If patient was tolerating TB treatment, eligible for SDI. |
| Physical examination | Nurses were instructed to screen out patients with any conditions that would require additional care. | Nurses were asked to use their clinical judgment; only conditions that posed risks for ART initiation were screened out. |
| Readiness | Any patient-reported barrier to uptake or adherence led to screening out. | Patient-reported barriers were not a criterion for screening out unless the study nurse considered them insurmountable. For example, patient concern about future transportation to the clinic was no longer a reason for screening out. |

*Further details about the SLATE II algorithm and comparison to SLATE I can be found in the previously published protocol paper [13].

TB = Tuberculosis

LAM = lipoarabinomannan

SDI = same-day initiation
