# Supplementary material for: A clinical algorithm for same-day HIV treatment initiation in settings with high TB symptom prevalence in South Africa: The SLATE II individually randomized clinical trial
Source: PLoS Med. 2020 Aug 27;17(8):e1003226. doi: 10.1371/journal.pmed.1003226 (PMC7451542; doi:10.1371/journal.pmed.1003226)
Supplement: S2 Table — (DOCX) [file pmed.1003226.s003.docx]

### **S2 Table. Effect measure modification by key variables**

| **Stratification variables** | **Outcome 1: Initiated ART≤ 7 days** | | | |  | **Outcome 2: Initiated ≤ 28 days and retained at 8 months** | | | |  |
| --- | --- | --- | --- | --- | --- | --- | --- | --- | --- | --- |
|  | **Standard arm** | **SLATE arm** | **RD (95% CI)** | **RR (95% CI)** | **RERI* (95% CI)** | **Standard arm** | **SLATE arm** | **RD**  **(95% CI)** | **RR**  **(95% CI)** | **RERI* (95% CI)** |
| Original analysis | 202/297 (68%) | 270/296 (91%) | 23%  (17-29%) | 1.34  (1.23-1.46) |  | 175/297 (59%) | 220/296 (74%) | 15%  (8-23%) | 1.26  (1.12-1.42) |  |
| *Study site* |  |  |  |  |  |  |  |  |  |  |
| Site 1 - COJ | 54/102 (53%) | 91/102 (89%) | 36%  (25-48%) | 1.69  (1.39-2.05) | Reference | 51/102 (50%) | 70/102 (69%) | 19%  (5-32%) | 1.37  (1.09-1.73) | Reference |
| Site 2 - Ekurhuleni | 79/107 (74%) | 97/105 (92%) | 18%  (9-28%) | 1.25  (1.10-1.42) | 0.86 (0.77-0.97) | 75/107 (70%) | 86/105 (82%) | 12%  (0-23%) | 1.17  (1.00-1.36) | 0.92 (0.80-1.06) |
| Site 3 - COJ | 69/88 (78%) | 82/89 (92%) | 14%  (3-24%) | 1.18  (1.04-1.33) | 0.70 (0.55-0.88) | 49/88 (56%) | 64/89 (72%) | 16% (2-30%) | 1.29  (1.03-1.62) | 0.94 (0.68-1.30) |
| *Sex* |  |  |  |  |  |  |  |  |  |  |
| Male | 75/113 (66%) | 96/107 (90%) | 24%  (13-34%) | 1.35  (1.17-1.56) | Reference | 71/113 (63%) | 81/107 (76%) | 13%  (1-25%) | 1.20  (1.01-1.44) | Reference |
| Female | 127/184 (69%) | 174/189 (92%) | 23%  (15-31%) | 1.33  (1.20-1.48) | 0.99 (0.85-1.21) | 104/184 (57%) | 139/189 (74%) | 17% (7-27%) | 1.30  (1.12-1.52) | 1.08 (0.85-1.37) |
| *CD4 count at enrolment* | |  |  |  |  |  |  |  |  |  |
| <200 cells | 66/107 (62%) | 88/106 (83%) | 21%  (10-33%) | 1.35  (1.13-1.60) | Reference | 62/107 (58%) | 78/106 (74%) | 16%  (3-28%) | 1.27  (1.04-1.55) | Reference |
| 200+ cells | 126/176 (72%) | 178/185 (96%) | 25%  (17-32%) | 1.34  (1.22-1.48) | 1.00 (0.99-1.00) | 103/176 (59%) | 139/185 (75%) | 16%  (7-26%) | 1.28 (1.11-1.49) | 1.00 (0.99-1.00) |
| *Age at enrolment* | |  |  |  |  |  |  |  |  |  |
| <35 years | 93/135 (69%) | 138/146 (95%) | 26%  (17-34%) | 1.37  (1.22-1.55) | Reference | 74/135 (55%) | 107/146 (73%) | 18%  (7-30%) | 1.34  (1.11-1.60) | Reference |
| 35+ years | 109/162 (67%) | 132/150 (88%) | 21%  (12-30%) | 1.31 (1.16 – 1.48) | 0.95 (0.80 -1.13) | 101/162 (62%) | 113/150 (75%) | 13%  (3-23%) | 1.21  (1.04-1.40) | 0.90 (0.71-1.14) |
| *Reason for visit* | |  |  |  |  |  |  |  |  |  |
| HIV test (diagnosis) | 81/110 (74%) | 102/108 (94%) | 21%  (12-30%) | 1.28  (1.14-1.45) | Reference | 67/110 (61%) | 80/108 (74%) | 13%  (1-25%) | 1.22  (1.01-1.47) | Reference |
| Other reason | 121/187 (65%) | 168/188 (89%) | 25%  (17-33%) | 1.38  (1.23-1.55) | 1.08 (0.91-1.27) | 108/187 (58%) | 140/188 (74%) | 17%  (7-26%) | 1.29  (1.11-1.50) | 1.06 (0.83-1.35) |
| *TB symptom at enrolment* | |  |  |  |  |  |  |  |  |  |
| ≥1 TB symptoms | 96/155 (62%) | 115/140 (82%) | 20%  (10-30%) | 1.33  (1.15-1.53) | Reference | 85/155 (55%) | 95/140 (68%) | 13%  (2-24%) | 1.24  (1.03-1.49) | Reference |
| No TB symptoms | 106/142 (75%) | 155/156 (99%) | 25%  (17-32%) | 1.33  (1.21-1.47) | 1.00 (0.84-1.19) | 90/142 (63%) | 125/156 (80%) | 17%  (7-27%) | 1.26  (1.09-1.47) | 1.02 (0.81-1.29) |

*RERI = relative excess risk due to interaction
