# Supplementary material for: A clinical algorithm for same-day HIV treatment initiation in settings with high TB symptom prevalence in South Africa: The SLATE II individually randomized clinical trial
Source: PLoS Med. 2020 Aug 27;17(8):e1003226. doi: 10.1371/journal.pmed.1003226 (PMC7451542; doi:10.1371/journal.pmed.1003226)
Supplement: S3 Table — (DOCX) [file pmed.1003226.s004.docx]

### **S3 Table. Viral suppression with 2-8 month window for test results***

| **Outcome** | **Standard arm (n=297)** | **Intervention arm (n=296)** | **Crude risk difference (95%CI)†** | **Crude relative risk (95% CI)†** |
| --- | --- | --- | --- | --- |
| Initiated ART ≤ 28 days and known to be virally suppressed ≤ 8 months** | 114 (38%) | 154 (52%) | 14% (6-22%) | 1.36 (1.13-1.62) |
| Initiated ART ≤ 28 days and not known to be virally suppressed ≤ 8 months | 61 (21%) | 66 (22%) | 1% (-5 to 8%) | 1.07 (0.79-1.45) |
| Known unsuppressed VL result | 15 (5%) | 15 (5%) | 0% (-4 to 4%) | 1.00 (0.50-2.02) |
| VL result not traced | 46 (16%) | 51 (17%) | 1% (-5 to 7%) | 1.09 (0.76-1.56) |
| Initiated ART ≤ 28 days but not retained in care | 68 (23%) | 57 (19%) | -4% (-10 to 3%) | 0.84 (0.62 -1.15) |
| No record of initiation ≤ 28 days | 54 (18%) | 19 (6%) | -11% (-17 to -7%) | 0.35 (0.21-0.58) |

*Window for viral load testing expanded to 2-8 months after study enrollment, rather than the 5-8 month window used in the primary analysis.

†Reference group: standard arm
