# Supplementary material for: A clinical algorithm for same-day HIV treatment initiation in settings with high TB symptom prevalence in South Africa: The SLATE II individually randomized clinical trial
Source: PLoS Med. 2020 Aug 27;17(8):e1003226. doi: 10.1371/journal.pmed.1003226 (PMC7451542; doi:10.1371/journal.pmed.1003226)
Supplement: S4 Table — (DOCX) [file pmed.1003226.s005.docx]

**S4 Table. Mortality reported during study follow up**

| **Study arm** | **Days between study enrollment and death** | **CD4 count at enrollment (cells/mm^3^)** | **Eligible for same-day initiation under SLATE II algorithm?** | **Initiated ART prior to death?** |
| --- | --- | --- | --- | --- |
| Standard | 42 | 530 | NA | Yes |
| Standard | 36 | 11 | NA | Yes |
| Standard | 72 | 93 | NA | Yes |
| Intervention | 101 | 19 | No | Yes |
| Standard | 27 | 2 | NA | No* |
| Intervention | 7 | 3 | Yes | Yes |
| Standard | 9 | 62 | NA | Yes |

NA, not applicable (standard arm patient)

*Note: In Table 3, this patient is included in the row labeled “no record of initiation ≤28 days,” and not in the row labeled “not retained-died,” which captures only patients who initiated ART ≤28 days but died before the 8-month study endpoint.
