# Supplementary material for: A clinical algorithm for same-day HIV treatment initiation in settings with high TB symptom prevalence in South Africa: The SLATE II individually randomized clinical trial
Source: PLoS Med. 2020 Aug 27;17(8):e1003226. doi: 10.1371/journal.pmed.1003226 (PMC7451542; doi:10.1371/journal.pmed.1003226)
Supplement: S5 Table — SLATE, Simplified Algorithm for Treatment Eligibility. (DOCX) [file pmed.1003226.s006.docx]

**S5 Table. Comparison of SLATE I and SLATE II results**

| **Outcome** | **SLATE I** | | | **SLATE II** | | |
| --- | --- | --- | --- | --- | --- | --- |
|  | **Standard arm (n=302)** | **Intervention arm**  **(n=298)** | **Crude risk difference (95% CI)** | **Standard arm (n=297)** | **Intervention arm**  **(n=296)** | **Crude risk difference (95% CI)** |
| Initiated in 0 days (same day) | 33 (11%) | 161 (54%) | 43%  (36- 50%) | 114 (38%) | 257 (87%) | 49%  (42-55%) |
| Initiated within 7 days | 114 (38%) | 193 (65%) | 27%  (19-35%) | 202 (68%) | 270 (91%) | 23%  (17-29%) |
| Initiated within 14 days | 170 (56%) | 207 (69%) | 13%  (6-20%) | 228 (77%) | 274 (93%) | 16% (10-21%) |
| Initiated within 28 days | 204 (68%) | 232 (78%) | 10%  (3-17%) | 243 (82%) | 277 (94%) | 12%  (7-17%) |
| Initiated within 90 days | 238 (79%) | 256 (86%) | 7%  (0 -11%) | 253 (85%) | 280 (95%) | 10%  (5-14%) |
| No record of initiation ≤90 days | 64 (21%) | 42 (14%) | -7%  (-13-1%) | 44 (15%) | 16 (5%) | -10%  (-14 -5%) |
| Initiated ART ≤ 28 days and retained in care at 8 months | 146 (48%) | 161 (54%) | 6%  (-2%-14%) | 175 (59%) | 220 (74%) | 15% (8 to 23%) |
| Initiated ART ≤ 28 days and known to be virally suppressed ≤ 8 months | 90 (30%) | 93 (31%) | ﻿1%  (-6-9%) | 94 (32%) | 130 (44%) | 12%  (5-20%) |
